# Supplementary figures and images for: A new mouse model of Charcot-Marie-Tooth 2J neuropathy replicates human axonopathy and suggest alteration in axo-glia communication
Source: PLoS Genet. 2022 Nov 9;18(11):e1010477. doi: 10.1371/journal.pgen.1010477 (PMC9707796; doi:10.1371/journal.pgen.1010477)

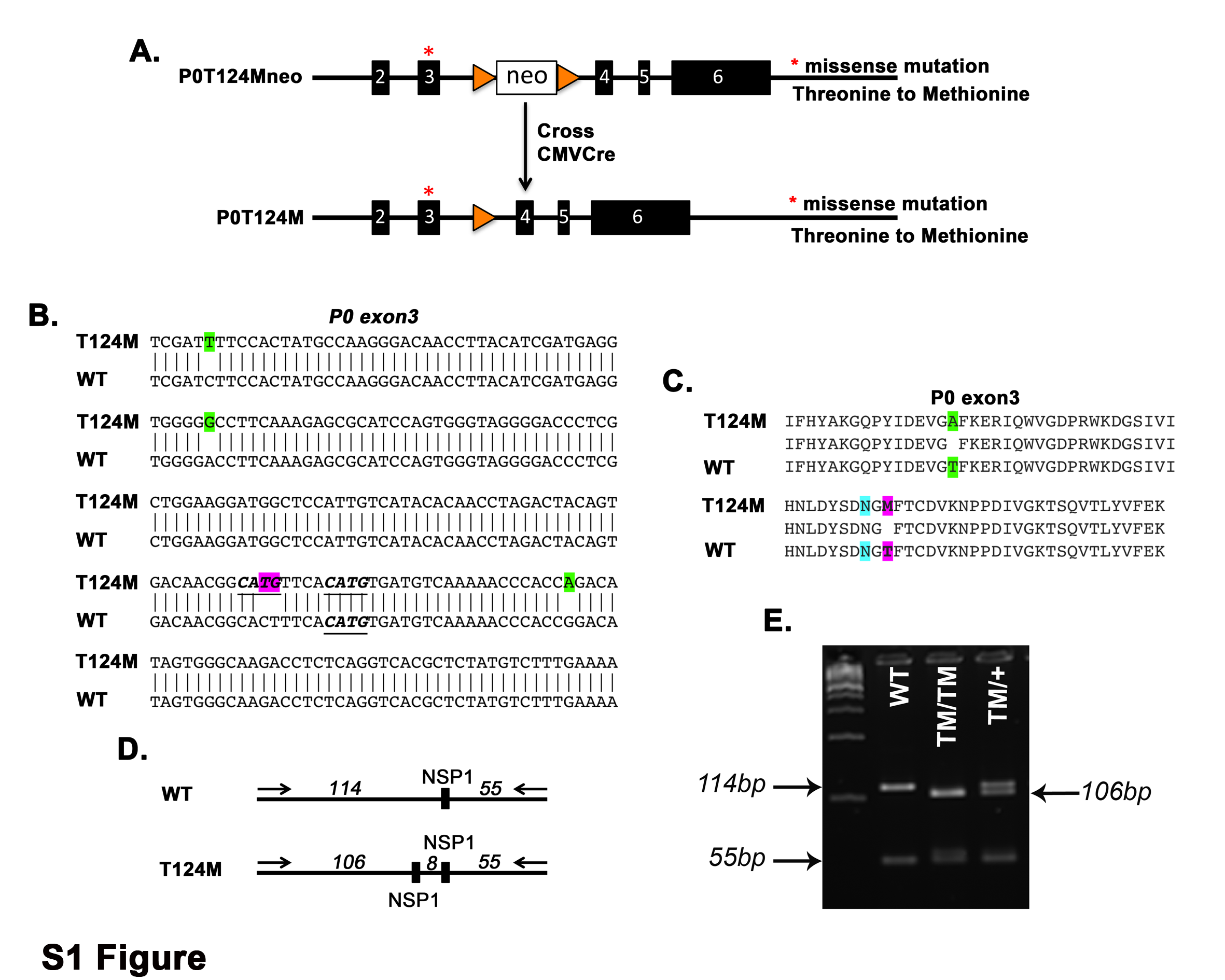

Supplement: S1 Fig — (A) The targeting construct to introduce the T124M mutation into exon 3 of the mouse Mpz gene is based on the construct used to engineer the R98C mouse. The neomycin resistance gene was removed by crossing the founder mice with CMV-Cre mice. (B and C) P0 mRNA from wild-type (WT) and MpzT124M/T124M (T124M) sciatic nerves was cloned and sequenced. Nucleotide (B) and amino acid (C) sequences (P0 exon 3) for the wild type (WT) and mutant (T124M) were compared. T124M substitution is highlighted in purple. Note how close the T124M mutation is to the N-glycosylation acceptor site N122 (highlighted in blue). Strain-specific neutral mutation is indicated in green. More details are provided in S1 Data. (D) MpzT124M mouse genotyping strategy. Introduction of T124M mutation generates a restriction recognition site for NSP1. This new site is located 8 bp from the 5′ end of a preexisting NSP1 restriction site. After amplification, DNA from the wild type (WT) is cut once by NSP1 to generate two fragments of 114 and 55 bp. DNA from the MpzT124M mutant is cut at two different sites by NSP1 to generate three fragments of 106, 8, and 55 bp. (E) Representative genotyping PCR of WT, MpzT124M/+, and MpzT124M/T124M. WT has a band at 114 bp. MpzT124M heterozygote exhibits the 114 bp band and the 106-bp mutant band. MpzT124M homozygote shows only one band at 106 bp. The three genotypes have a band at 55 bp; 8 bp is too small to be detected. (TIF) [file pgen.1010477.s001.tif]

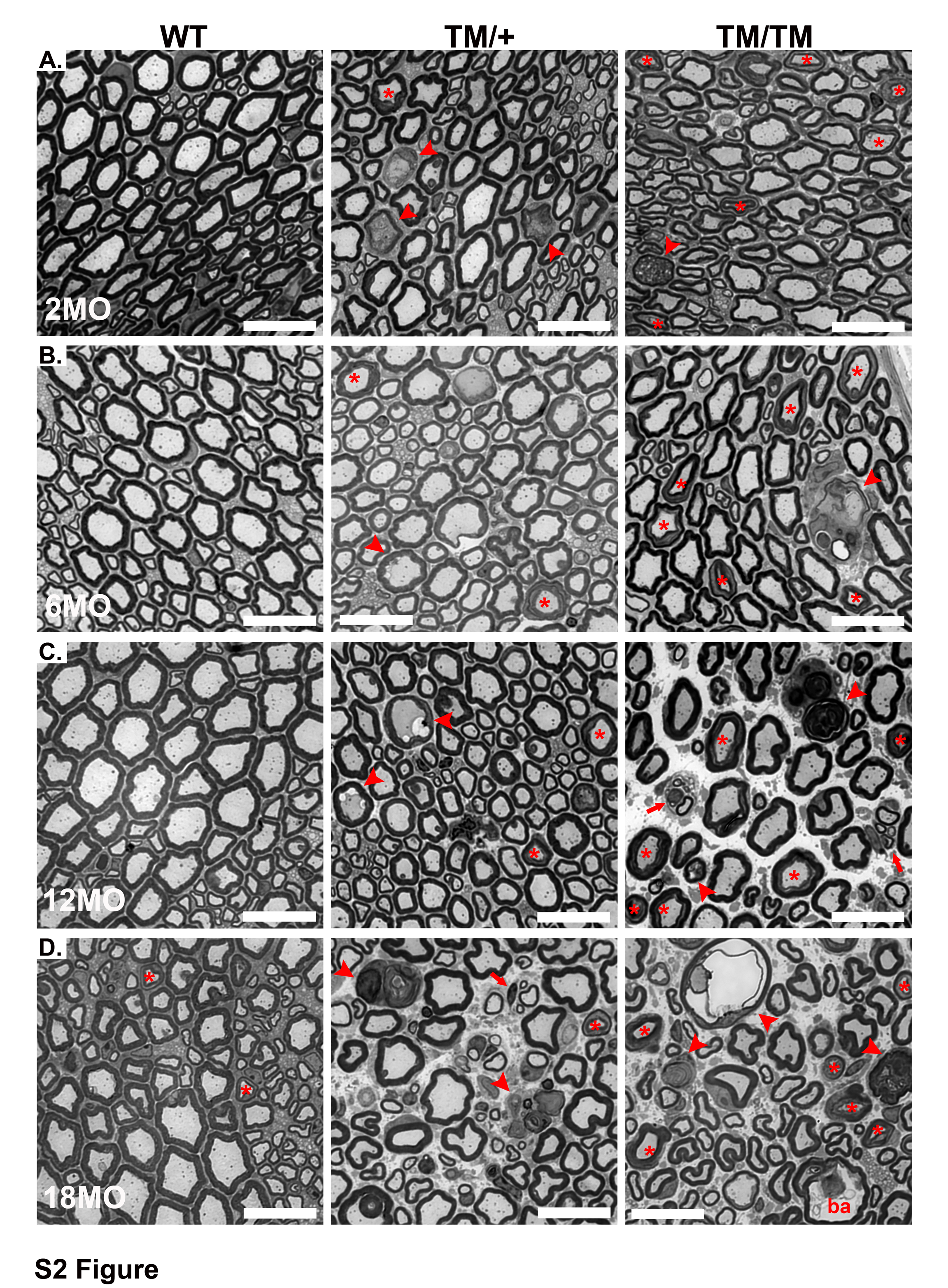

Supplement: S2 Fig — Representative images of transverse semithin sections of sciatic nerves stained with toluidine blue from wild-type (WT) and MpzT124M/+ (TM/+) and MpzT124M/T124M (TM/TM) mice at 2 (A), 6 (B), 12 (C), and 18 (D) months of age. Arrowheads indicate degenerative figures, arrows indicate regenerative axons, asterisks indicate Schmidt-Lanterman incisures (SLI), and “ba” indicates a myelin balloon. Scale bars: 20 μm. (TIF) [file pgen.1010477.s002.tif]

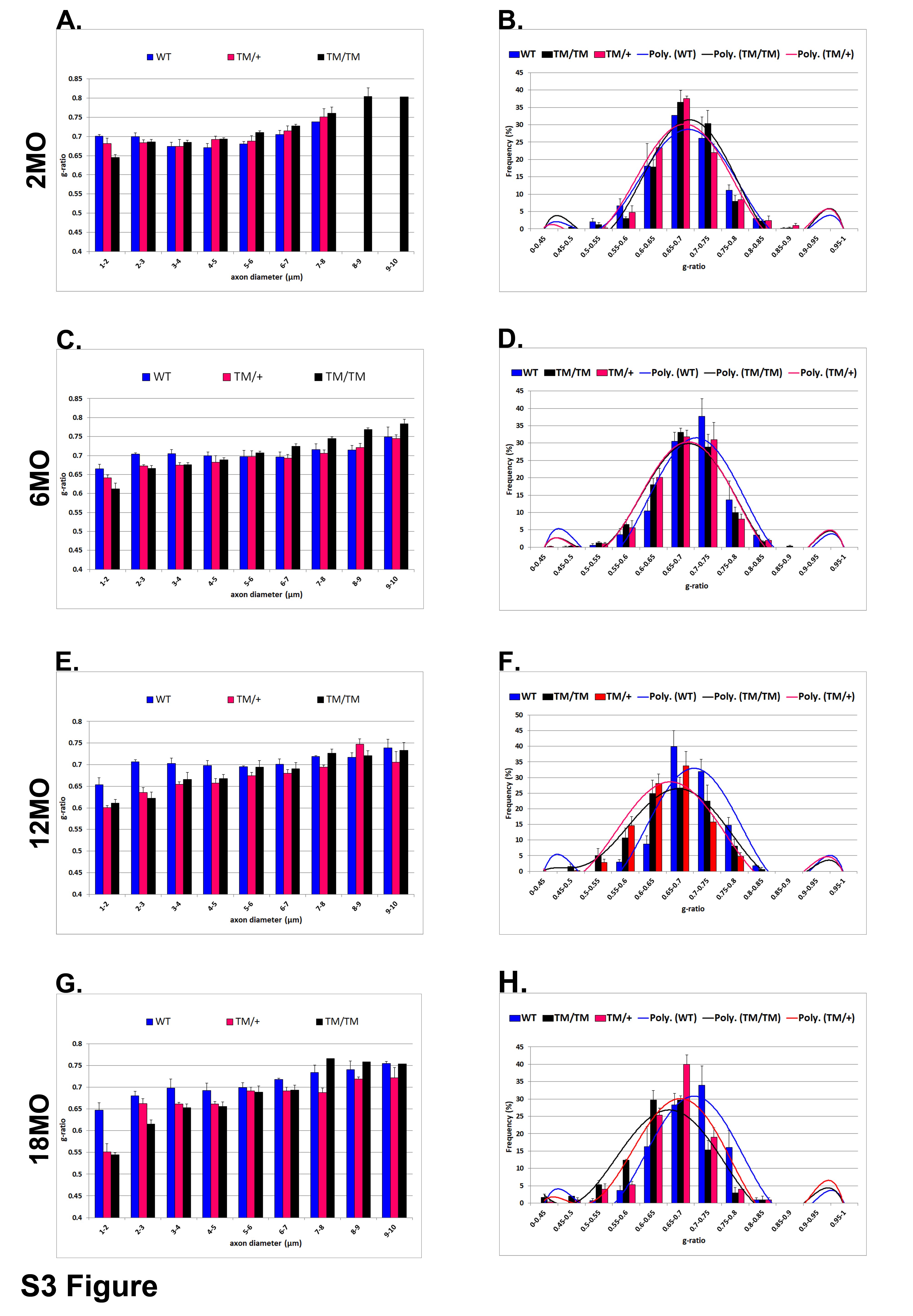

Supplement: S3 Fig — From 2 (A) to 6 (C) months of age, we observed similar myelin thickness between WT and MpzT124M nerves, whatever axon caliber. Relative frequency of g-ratio is equally distributed among WT and MpzT124M mutants at 2 (B) and 6 (D) months of age. g-ratio as a function of axonal diameter shows hypermyelination of small fibers in MpzT124M mice compared to that in WT mice at 12 (E) and 18 (G) months of age. Relative frequency of g-ratio is shifted toward smaller g-ratio values in MpzT124M fibers at 12 (F) and 18 (H) months of age. (TIF) [file pgen.1010477.s003.tif]

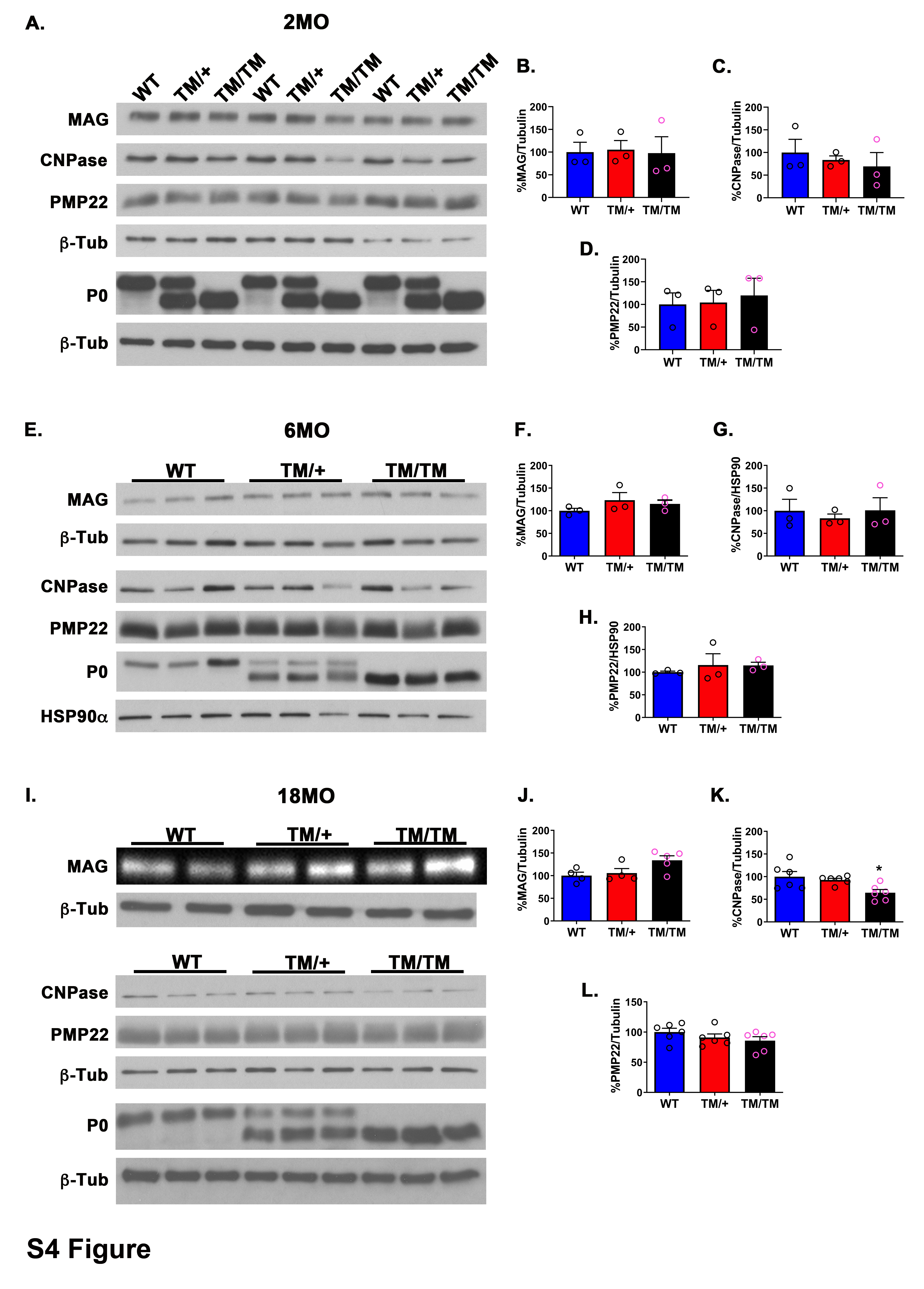

Supplement: S4 Fig — Western blot analysis for wild-type (WT) and MpzT124M/+ (TM/+) and MpzT124M/T124M (TM/TM) mice at 2 (A), 6 (E), and 18 (I) months of age. Blots were probed with CNP, PMP22, and P0 antibodies. β-Tubulin and HSP90α were used as loading controls. Densitometric quantification did not reveal an alteration of MAG (B, F, and J) or PMP22 (D, H, and L) expression. CNP expression was reduced in MpzT124M/T124M mice at 18 months of age (K) [F (2, 15) = 5.534, p = 0.0158] but not at younger ages (C and G). T124M mutation alters P0 migration. n (animals) ≥ 3 per genotype. *p < 0.05 by multiple-comparisons Tukey’s post hoc tests after one-way ANOVA (B to D, F to H, and J to L). Graphs indicate means ± SEMs. (TIF) [file pgen.1010477.s004.tif]

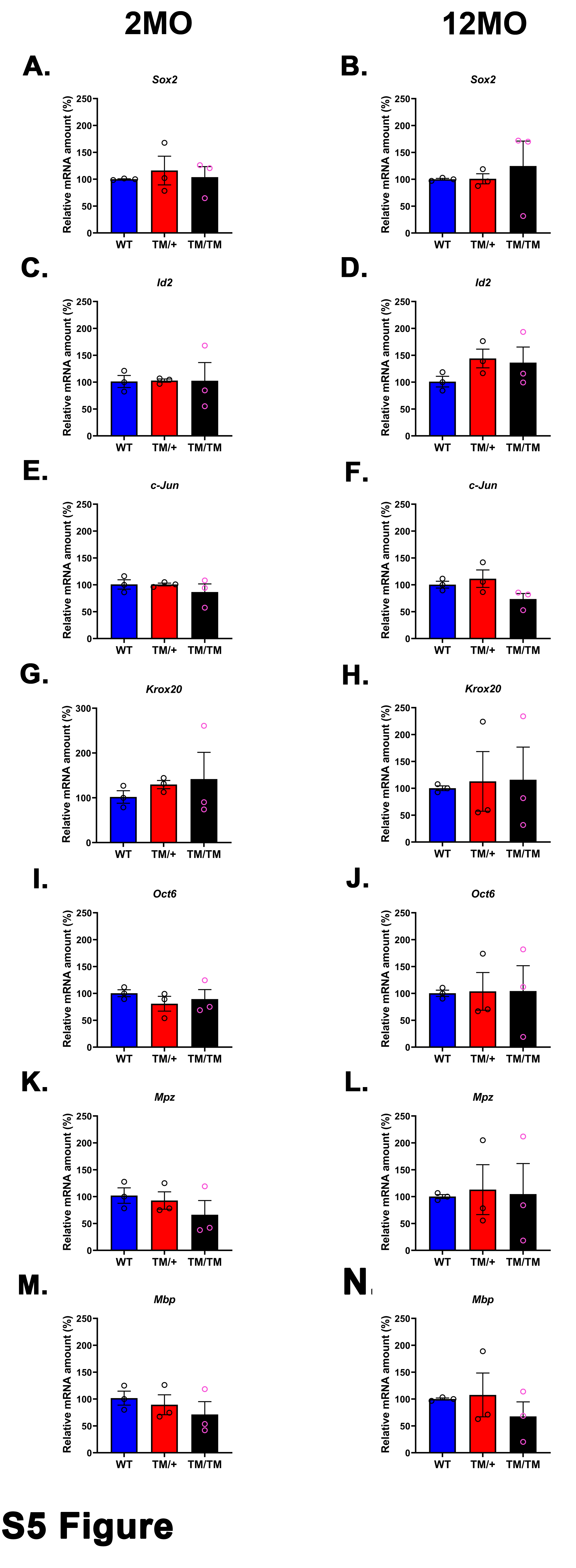

Supplement: S5 Fig — Total RNA was extracted from wild-type (WT), MpzT124M/+ (TM/+) and MpzT124M/T124M (TM/TM) sciatic nerves at 2 and 12 months of age. Quantitative real-time PCR experiments were performed using primers recognizing Sox2 (A and B), Id2 (C and D), c-Jun (E and F), Krox20 (G and H), Oct6 (I and J), Mpz (K and L) and Mbp (M and N). The qRT-PCR was normalized using 18S RNA. n (animals) ≥ 3 per genotype. Multiple-comparisons Tukey’s post hoc tests after one-way ANOVA. Graphs indicate means ± SEMs. (TIF) [file pgen.1010477.s005.tif]

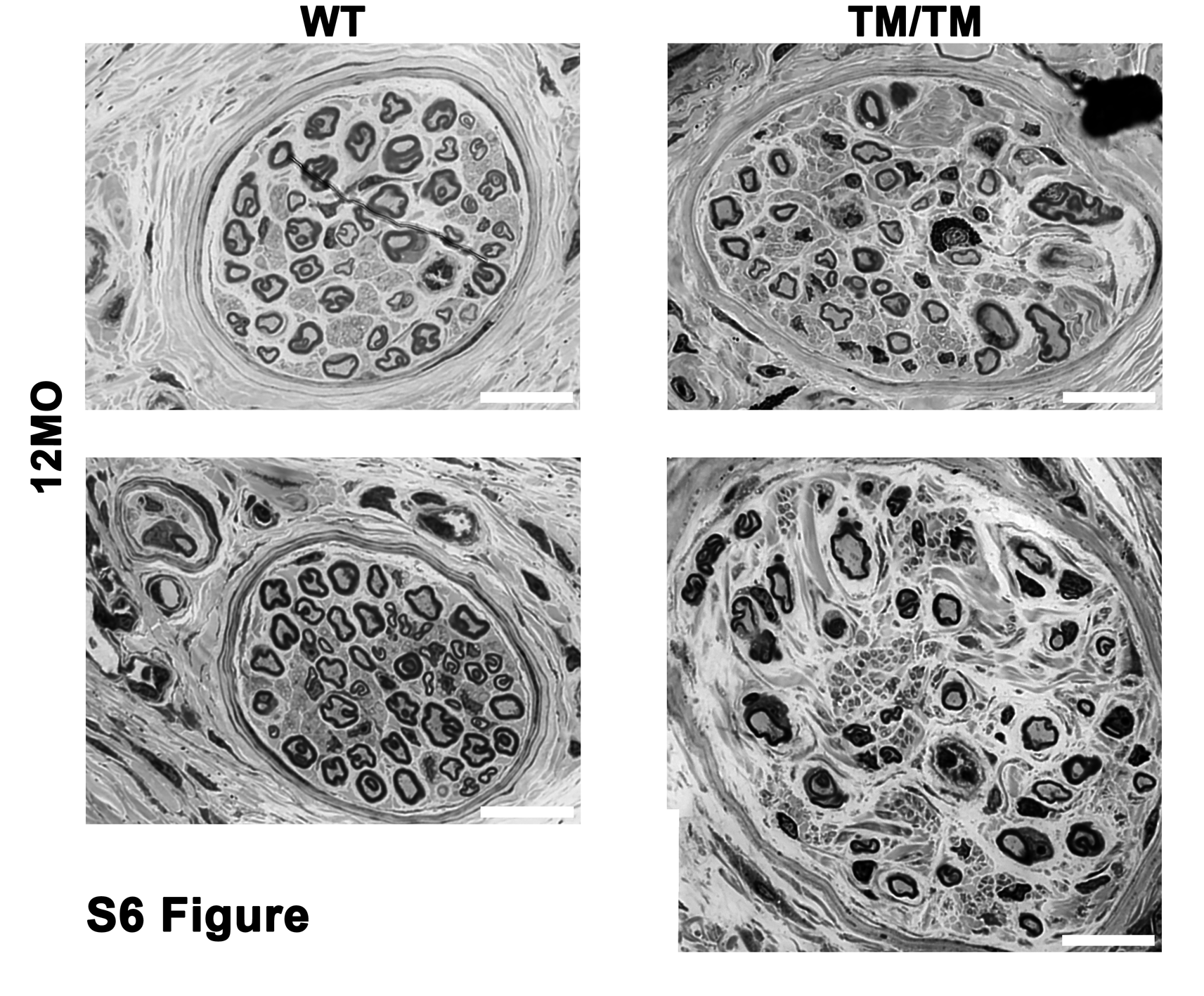

Supplement: S6 Fig — Representative images of transverse semithin sections of digital nerves stained with toluidine blue from two wild-type (WT) and two MpzT124M/T124M (TM/TM) mice at 12 months of age in FVB/N background. Scale bars: 20 μm. (TIF) [file pgen.1010477.s006.tif]

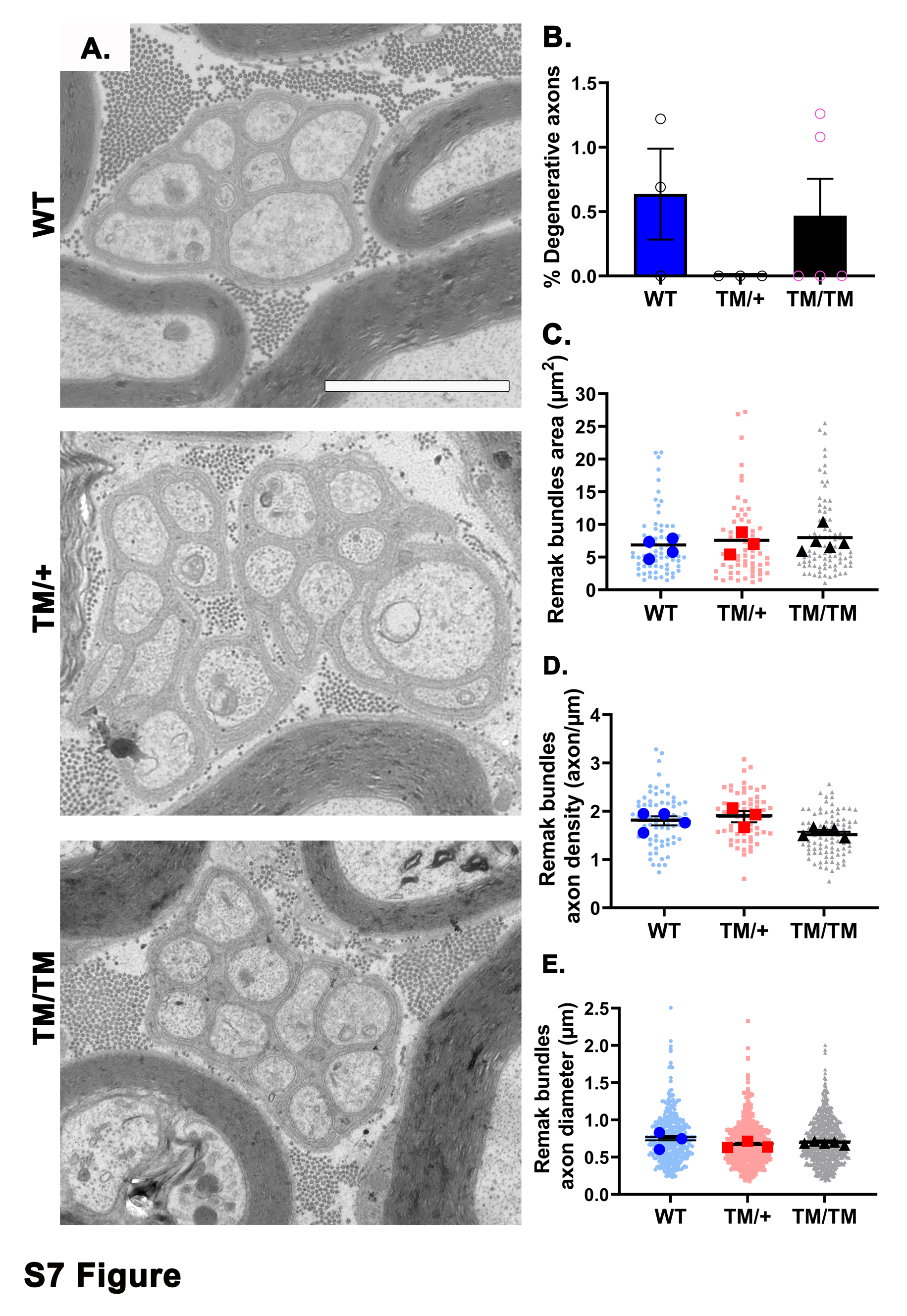

Supplement: S7 Fig — (A) Representative electron micrograph of Remak bundles of wild-type (WT), MpzT124M/+ (TM/+) and MpzT124M/T124M (TM/TM) sciatic nerves. Scale bar: 2μm. As in WT, in MpzT124M axons are uniformly ensheathed by SC cytoplasm. (B) Degenerating axons were not observed in MpzT124M mutants Remak bundles. (C) Quantification of Remak bundles area (μm2). (D) Quantification of Remak bundles axon density (axon per μm). (E) Quantification of axon diameters size (μm). n (animals) ≥ 3 per genotype; at least 390 axons per genotype were quantified. Multiple-comparisons Tukey’s post hoc tests after one-way ANOVA (B) and Nested one-way ANOVA (C, D, E). Graphs indicate means ± SEMs. (TIF) [file pgen.1010477.s007.tif]

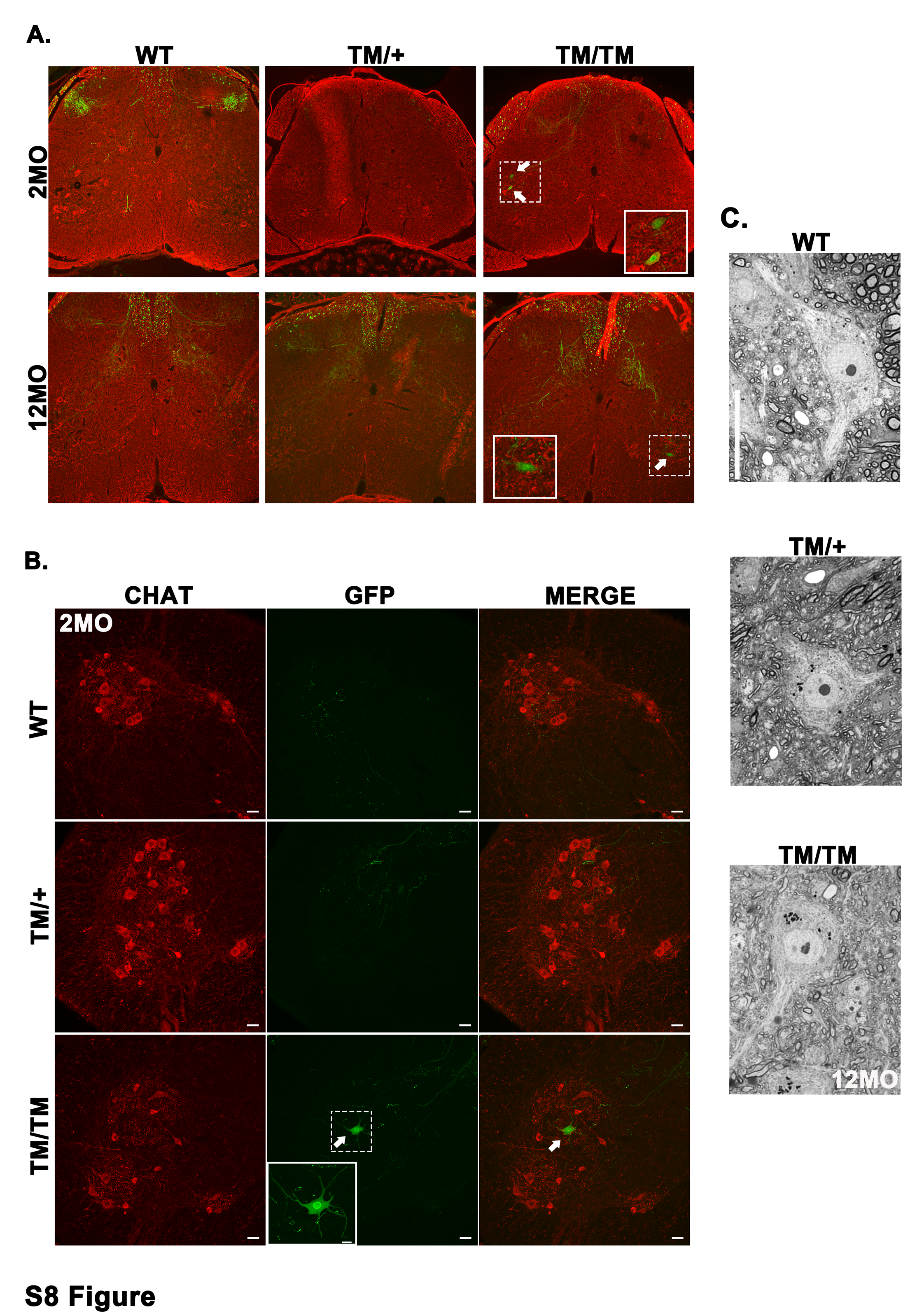

Supplement: S8 Fig — Representative confocal microscopy images of spinal cords sections (L3-L5) from 2 and 12-month-old wild-type (WT), MpzT124M/+ (TM/+) and MpzT124M/T124M (TM/TM)–ATF3-GFP mice stained for TuJ1 (red) (A), choline acetyltransferase (CHAT; motoneurons) (red) (B) and ATF3-GFP (green). Scale bars: 40 μm. High-magnification insets show motoneuron expressing GFP under ATF3 promoter control. (C) Representative images of transverse semithin sections of lumbar spinal cords stained with toluidine blue from WT, MpzT124M/+ and MpzT124M/T124M mice at 12 months of age. n (animals) ≥ 3 per genotype. (TIF) [file pgen.1010477.s008.tif]

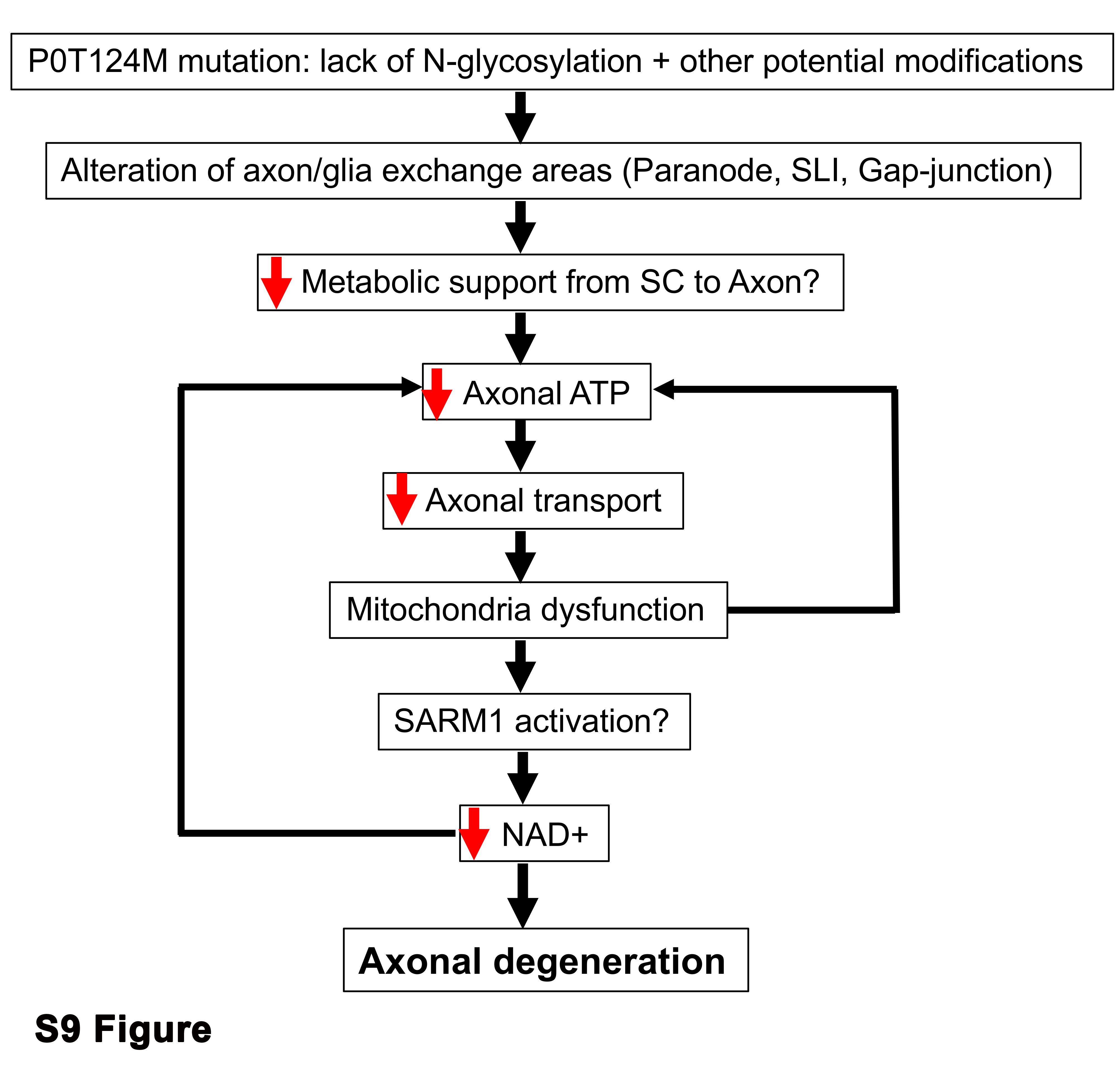

Supplement: S9 Fig — P0T124M mutation impedes N-glycosylation and is responsible for additional P0 modifications. Axon–glia exchange areas (paranodes, Schmidt-Lanterman incisures [SLI], and gap junctions) are altered in MpzT124M mutants and could lead to deficient transport of metabolites from Schwann cells (SC) to axons. Lack of SC support deprives axons of ATP. Axonal transport is slowed, leading to mitochondrial fragmentation, aggregation, and degeneration. Damaged mitochondria are not able to produce ATP, inducing a vicious cycle. Mitochondrial stress could activate SARM1, which cleaves NAD+. NAD+ depletion leads to axonal degeneration. (TIF) [file pgen.1010477.s009.tif]
