# Supplementary material for: A new mouse model of Charcot-Marie-Tooth 2J neuropathy replicates human axonopathy and suggest alteration in axo-glia communication
Source: PLoS Genet. 2022 Nov 9;18(11):e1010477. doi: 10.1371/journal.pgen.1010477 (PMC9707796; doi:10.1371/journal.pgen.1010477)
Supplement: S1 Data — (DOCX) [file pgen.1010477.s013.docx]

**Sciatic nerve 32717 clone 1 nucleotide sequence**

TTTCCTTAGATCATGCTCGAGCGGCCGCCAGTGTGATGGATATCTGCAGAATTCGGCTTATGGCTCCCGGGGCTCCCTCCTCCAGCCCCAGCCCTATCCTGGCTGCCCTGCTCTTCTCTTCTTTGGTGCTGTCTCCAGCCCTGGCCATTGTGGTTTACACGGACAGGGAAATCTATGGTGCTGTGGGCTCCCAGGTGACCCTGCACTGCTCCTTCTGGTCCAGTGAATGGGTCTCAGATGACATCTCTTTTACCTGGCGCTACCAGCCTGAAGGGGGCCGAGATGCCATTTCGATTTTCCACTATGCCAAGGGACAACCTTACATCGATGAGGTGGGGGCCTTCAAAGAGCGCATCCAGTGGGTAGGGGACCCTCGCTGGAAGGATGGCTCCATTGTCATACACAACCTAGACTACAGTGACAACGGCATGTTCACATGTGATGTCAAAAACCCACCAGACATAGTGGGCAAGACCTCTCAGGTCACGCTCTATGTCTTTGAAAAAGTGCCCACTAGGTATGGGGTGGTGTTGGGAGCAGTGATCGGGGGCATCCTCGGGGTGGTGCTGTTGCTGCTGTTGCTCTTCTACCTGATTCGGTACTGCTGGCTGCGCAGGCAGGCTGCCCTGCAGAGAAGGCTCAGTGCCATGGAGAAGGGGAGATTTCACAAATCTTCGAAGGACTCCTCGAAGCGAGGGCGGCAGACGCCAGTGCTGTATGCCATGCTGGACCACAGCCGAAGCACCAAAGCTGCCAGTGAGAAGAAATCAAAAGGGCTGGGGGAGTCTCGCAAGGATAAGAAAATAGAAGCCGAATTCCAGCACACTGGCGGCCGTTACTAGTGGATCCGAGCTCGGTACCAAGCTTGGGCGTAATCATGGTCATAGCTGTTTCCTGTGTGAAATTGTTATCCGCTCACAATTCCACACAACATACGAGCCGGAAGCATAAAGTGTAAAGCCTGGGGTGCCTAATGAGTGAGCTAACTCACATTAATTTGCGTTGCGCTCACTGCCGCTTTCCAGTCGGAAACCTGTCGTGCCAGCTGCATATGATCGGCCACGCGCGGGGAGAGGCGTTGCGTATGGCGCCTCTTCGCTCTCGCTTCACTGAACTCGCTGGCGCTCGGTCGATTCCGGCCTGCGCGGCCGAGACG

Start codon Stop codon

**1. Standard nucleotide blast NCBI**

Mus musculus myelin protein zero (Mpz), mRNA

Sequence ID: [ref|NM_008623.4|](http://www.ncbi.nlm.nih.gov/nucleotide/162139828?report=genbank&log$=nuclalign&blast_rank=2&RID=FX566W2M01R)Length: 1993Number of Matches: 1

Related Information

[Gene](http://www.ncbi.nlm.nih.gov/gene?term=162139828%5bNUID%5d&RID=FX566W2M01R&log$=genealign&blast_rank=2)-associated gene details

[GEO Profiles](http://www.ncbi.nlm.nih.gov/geoprofiles/?LinkName=nucleotide_geoprofiles&from_uid=162139828&RID=FX566W2M01R&log$=geoalign&blast_rank=2)-microarray expression data

[Map Viewer](http://www.ncbi.nlm.nih.gov/mapview/map_search.cgi?direct=on&gbgi=162139828&THE_BLAST_RID=FX566W2M01R&log$=mapalign&blast_rank=2)-aligned genomic context

Range 1: 167 to 910[GenBank](http://www.ncbi.nlm.nih.gov/nucleotide/162139828?report=genbank&log$=nuclalign&blast_rank=2&RID=FX566W2M01R&from=167&to=910)[Graphics](http://www.ncbi.nlm.nih.gov/nuccore/162139828?report=graph&rid=FX566W2M01R%5b162139828%5d&tracks=%5bkey:sequence_track,name:Sequence,display_name:Sequence,id:STD1,category:Sequence,annots:Sequence,ShowLabel:true%5d%5bkey:gene_model_track,CDSProductFeats:false%5d%5bkey:alignment_track,name:other%20alignments,annots:NG%20Alignments%7CRefseq%20Alignments%7CGnomon%20Alignments%7CUnnamed,shown:false%5d&v=130:947&appname=ncbiblast&link_loc=fromHSP) Next Match Previous Match

| Alignment statistics for match #1 | | | | |
| --- | --- | --- | --- | --- |
| **Score** | **Expect** | **Identities** | **Gaps** | **Strand** |
| 1310 bits(1452) | 0.0 | 737/744(99%) | 0/744(0%) | Plus/Plus |

Query 1 ATGGCTCCCGGGGCTCCCTCCTCCAGCCCCAGCCCTATCCTGGCTGCCCTGCTCTTCTCT 60

||||||||||||||||||||||||||||||||||||||||||||||||||||||||||||

Sbjct 167 ATGGCTCCCGGGGCTCCCTCCTCCAGCCCCAGCCCTATCCTGGCTGCCCTGCTCTTCTCT 226

Query 61 TCTTTGGTGCTGTCTCCAGCCCTGGCCATTGTGGTTTACACGGACAGGGAAATCTATGGT 120

||||||||||| ||||||||||||||||||||||||||||||||||||||||||||||||

Sbjct 227 TCTTTGGTGCTCTCTCCAGCCCTGGCCATTGTGGTTTACACGGACAGGGAAATCTATGGT 286

Query 121 GCTGTGGGCTCCCAGGTGACCCTGCACTGCTCCTTCTGGTCCAGTGAATGGGTCTCAGAT 180

|| |||||||||||||||||||||||||||||||||||||||||||||||||||||||||

Sbjct 287 GCCGTGGGCTCCCAGGTGACCCTGCACTGCTCCTTCTGGTCCAGTGAATGGGTCTCAGAT 346

Query 181 GACATCTCTTTTACCTGGCGCTACCAGCCTGAAGGGGGCCGAGATGCCATT**TCGATTTTC** 240

|||||||||||||||||||||||||||||||||||||||||||||||||||||||| |||

Sbjct 347 GACATCTCTTTTACCTGGCGCTACCAGCCTGAAGGGGGCCGAGATGCCATT**TCGATCTTC** 406

Query 241 **CACTATGCCAAGGGACAACCTTACATCGATGAGGTGGGGGCCTTCAAAGAGCGCATCCAG** 300

||||||||||||||||||||||||||||||||||||||| ||||||||||||||||||||

Sbjct 407 **CACTATGCCAAGGGACAACCTTACATCGATGAGGTGGGGACCTTCAAAGAGCGCATCCAG** 466

Query 301 **TGGGTAGGGGACCCTCGCTGGAAGGATGGCTCCATTGTCATACACAACCTAGACTACAGT** 360

||||||||||||||||||||||||||||||||||||||||||||||||||||||||||||

Sbjct 467 **TGGGTAGGGGACCCTCGCTGGAAGGATGGCTCCATTGTCATACACAACCTAGACTACAGT** 526

Query 361 **GACAACGGCATGTTCACATGTGATGTCAAAAACCCACCAGACATAGTGGGCAAGACCTCT** 420

|||||||||| |||||||||||||||||||||||||| |||||||||||||||||||||

Sbjct 527 **GACAACGGCACTTTCACATGTGATGTCAAAAACCCACCGGACATAGTGGGCAAGACCTCT** 586

Query 421 **CAGGTCACGCTCTATGTCTTTGAAAA**AGTGCCCACTAGGTATGGGGTGGTGTTGGGAGCA 480

||||||||||||||||||||||||||||||||||||||||||||||||||||||||||||

Sbjct 587 **CAGGTCACGCTCTATGTCTTTGAAAA**AGTGCCCACTAGGTATGGGGTGGTGTTGGGAGCA 646

Query 481 GTGATCGGGGGCATCCTCGGGGTGGTGCTGTTGCTGCTGTTGCTCTTCTACCTGATTCGG 540

||||||||||||||||||||||||||||||||||||||||||||||||||||||||||||

Sbjct 647 GTGATCGGGGGCATCCTCGGGGTGGTGCTGTTGCTGCTGTTGCTCTTCTACCTGATTCGG 706

Query 541 TACTGCTGGCTGCGCAGGCAGGCTGCCCTGCAGAGAAGGCTCAGTGCCATGGAGAAGGGG 600

||||||||||||||||||||||||||||||||||||||||||||||||||||||||||||

Sbjct 707 TACTGCTGGCTGCGCAGGCAGGCTGCCCTGCAGAGAAGGCTCAGTGCCATGGAGAAGGGG 766

Query 601 AGATTTCACAAATCTTCGAAGGACTCCTCGAAGCGAGGGCGGCAGACGCCAGTGCTGTAT 660

||||||||||||||||||||||||||||||||||||||||||||||||||||||||||||

Sbjct 767 AGATTTCACAAATCTTCGAAGGACTCCTCGAAGCGAGGGCGGCAGACGCCAGTGCTGTAT 826

Query 661 GCCATGCTGGACCACAGCCGAAGCACCAAAGCTGCCAGTGAGAAGAAATCAAAAGGGCTG 720

||||||||||||||||||||||||||||||||||||||||||||||||||||||||||||

Sbjct 827 GCCATGCTGGACCACAGCCGAAGCACCAAAGCTGCCAGTGAGAAGAAATCAAAAGGGCTG 886

Query 721 GGGGAGTCTCGCAAGGATAAGAAA 744

||||||||||||||||||||||||

Sbjct 887 GGGGAGTCTCGCAAGGATAAGAAA 910

Neutral mutation du to strain specificity T124M mutation **Exon3**

**Amino Acid sequence translation from 32717-clone1 nucleotide sequence**

MAPGAPSSSPSPILAALLFSSLVLSPALAIVVYTDREIYGAVGSQVTLHCSFWSSEWVSDDISFTWRYQPEGGRDAISIFHYAKGQPYIDEVGAFKERIQWVGDPRWKDGSIVIHNLDYSDNGMFTCDVKNPPDIVGKTSQVTLYVFEKVPTRYGVVLGAVIGGILGVVLLLLLLFYLIRYCWLRRQAALQRRLSAMEKGRFHKSSKDSSKRGRQTPVLYAMLDHSRSTKAASEKKSKGLGESRKDKK

**Amino Acid sequence from P0 *mus musculus* C57B6 NM_008623**

MAPGAPSSSPSPILAALLFSSLVLSPALAIVVYTDREIYGAVGSQVTLHCSFWSSEWVSDDISFTWRYQPEGGRDAISIFHYAKGQPYIDEVGTFKERIQWVGDPRWKDGSIVIHNLDYSDNGTFTCDVKNPPDIVGKTSQVTLYVFEKVPTRYGVVLGAVIGGILGVVLLLLLLFYLIRYCWLRRQAALQRRLSAMEKGRFHKSSKDSSKRGRQTPVLYAMLDHSRSTKAASEKKSKGLGESRKDKK

**2. Emboss water protein alignment**

Aligned_sequences: 2 # 1: EMBOSS_001 # 2: EMBOSS_001 # Matrix: EBLOSUM62 # Gap_penalty: 10.0 # Extend_penalty: 0.5 # # Length: 248 # Identity:246/248 (99.2%) # Similarity: 246/248 (99.2%) # Gaps: 0/248 ( 0.0%) # Score: 1283.0


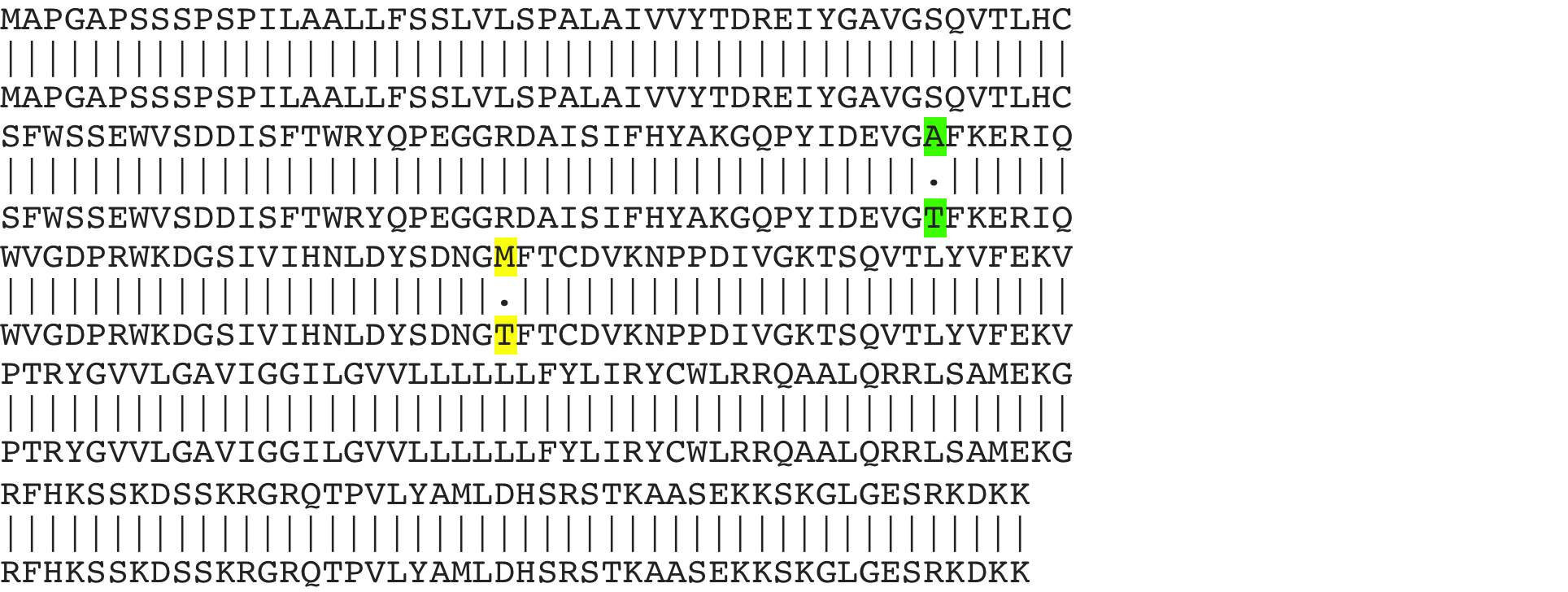


Neutral mutation du to strain specificity T124M mutation

**3. Standard protein blast NCBI**

**myelin protein P0 isoform L-MPZ precursor [Mus musculus]**

**Sequence ID:**[**NP_001302429.1**](https://www.ncbi.nlm.nih.gov/protein/NP_001302429.1?report=genbank&log$=protalign&blast_rank=1&RID=1BZFZZCA014)**Length: 312Number of Matches: 1**

Identities:246/248(99%), Positives: 246/248(99%), Gaps: 0/248(0%)

MAPGAPSSSPSPILAALLFSSLVLSPALAIVVYTDREIYGAVGSQVTLHCSFWSSEWVSD MAPGAPSSSPSPILAALLFSSLVLSPALAIVVYTDREIYGAVGSQVTLHCSFWSSEWVSD MAPGAPSSSPSPILAALLFSSLVLSPALAIVVYTDREIYGAVGSQVTLHCSFWSSEWVSD

DISFTWRYQPEGGRDAISIFHYAKGQPYIDEVGAFKERIQWVGDPRWKDGSIVIHNLDYS DISFTWRYQPEGGRDAISIFHYAKGQPYIDEVG FKERIQWVGDPRWKDGSIVIHNLDYS DISFTWRYQPEGGRDAISIFHYAKGQPYIDEVGTFKERIQWVGDPRWKDGSIVIHNLDYS

DNGMFTCDVKNPPDIVGKTSQVTLYVFEKVPTRYGVVLGAVIGGILGVVLLLLLLFYLIR DNG FTCDVKNPPDIVGKTSQVTLYVFEKVPTRYGVVLGAVIGGILGVVLLLLLLFYLIR DNGTFTCDVKNPPDIVGKTSQVTLYVFEKVPTRYGVVLGAVIGGILGVVLLLLLLFYLIR

YCWLRRQAALQRRLSAMEKGRFHKSSKDSSKRGRQTPVLYAMLDHSRSTKAASEKKSKGL YCWLRRQAALQRRLSAMEKGRFHKSSKDSSKRGRQTPVLYAMLDHSRSTKAASEKKSKGL YCWLRRQAALQRRLSAMEKGRFHKSSKDSSKRGRQTPVLYAMLDHSRSTKAASEKKSKGL

GESRKDKK

GESRKDKK

GESRKDKK

Neutral mutation du to strain specificity T124M mutation
