# Supplementary material for: A new mouse model of Charcot-Marie-Tooth 2J neuropathy replicates human axonopathy and suggest alteration in axo-glia communication
Source: PLoS Genet. 2022 Nov 9;18(11):e1010477. doi: 10.1371/journal.pgen.1010477 (PMC9707796; doi:10.1371/journal.pgen.1010477)
Supplement: Attachment — Submitted filename: Point by point reply.pdf [file pgen.1010477.s015.pdf]

Point-by point response to the reviewers' comments.

To the editors and the reviewers:
We would like to thank you for the positive and helpful evaluation of our manuscript. Our responses to the suggestions for improvements are indicated below and resulted in the addition of 3 new figures and modification of 10 figures, as detailed below. All resulting changes in the manuscript are typed in red.

Reviewer #1:

The authors describe a mouse model of the neuropathy (CMT2J) resulting from the T124M MPZ mutation. They provide convincing evidence for a predominantly axonal phenotype, as observed in human CMT2J patients. The paper is well written. It provides novel, interesting insight into the spectrum of MPZ functions and adds to our understanding of pathomechanisms of axonal and demyelinating neuropathies in general. The authors present a large body of sound data; still, in order to provide sufficient support for the hypotheses raised, a few points still need to be addressed in a revised version of the manuscript. "P0T124M mutation perturbs axon-glia interactions": This should be examined in more detail by EM, specifically by looking for abnormal Schwann cell-axon networks and related alterations.

Following the reviewer suggestion, we have now analyzed in more detail by EM the nodal and paranodal areas. Consistently with the fact that nodes were significantly wider in *MPZ*$^{T124M/T124M}$ nerves as compared to WT using immunohistochemistry for Caspr, we found that many *MPZ*$^{T124M/T124M}$ paranodes presented structural alteration with many paranodal loops largely disorganized, while WT loops were generally better organized although some alterations where sometimes found also in WT, possibly due to fixation issues. These observations have now been added to the txt (Line 444 to 451) and in Figure 5.

"Pathogenesis of P0T124M does not involve the unfolded protein response": Was there ultrastructural evidence of ER stress and/or altered autophagy in Schwann cells? If not, please state explicitly that this was absent.

In previous published work we have shown that activation of the UPR is a common mechanism in demyelinating CMT1B, and some data suggested that the UPR could play a part also in late onset CMT1B/CMT2J (Bai et al., 2018). This promped us to test the activation of the pathway also in the T124M mice. As explained, no activation was detected via qPCR and WB. Consistently, we could not see any ultrastructural evidence of ER stress. This is now stated in the text (line 428 to 429) and shown in figure 4J.

Axonal transport and mitochondrial disruption in T124M mice": Mitochondria / axoplasmic reticulum contacts should be examined. In addition, alterations of the axonal cytoskeleton (intraaxonal filamentous aggregates, altered microtubules, etc.) are of interest. Please address unmyelinated nerve fiber pathology.

We have collected evidence of axonal transport and mitochondrial defect in T124M such as: dramatic decrease of acetylated tubulin level, alteration of dynein and kinesin level, reduction of

TCA enzymes expression and presence of oxidative stress. However, these and other results concerning mitochondria bioenergetic dysfunction, mitochondria transport and their relationship with endoplasmic reticulum will be the topic of a following paper.

We have now carefully analyzed unmyelinated fibers structure, and showed that the T124M mutation does not affect Remak bundels, that appear properly formed and maintained. These observations are now included in the text (line 310-321) and in Supplementary Figure 7.

Spinal cord alpha motor neurons did not appear to be reduced in number. But did they should any cytological abnormalities?

Following the reviewer's suggestion we have now better characterized motor neurons morphology. As shown now in Fig. 3N and in Supplementary figure 8C, we could not detect any significant cytological alteration in motor neurons form T124M mice.

Did you examine DRG neurons?

We attempted to examine DRG neurons as well via immunofluorescence. Unfortunately, in DRG soma, ATF3-GFP background was too high to obtain reliable results. As such we currently cannot comment on DRG neurons number and/or morphology.

<u>Reviewer #2:</u>

Mutations in the peripheral myelin protein-zero (MPZ) result in peripheral neuropathy (Charcot Marie Tooth Disease) but can manifest itself in a range of distinct clinical pictures depending on the exact mutation. The missense mutation MPZ(T124M) is associated with CMT2J a form of CMT that is characterized by severe axonopathy but with surprisingly little evidence of demyelination, dismyelination or hypomyelination. To understand the patho-mechanism of this form of CMT and that of other forms of CMT primarily characterized by an axonopathy, the authors have generated a mouse model in which the exact missense mutation T124M is engineered in the mouse MPZ gene. The paper describes the meticulous characterization of mice heterozygous and homozygous for this mutation and find that it largely recapitulates the human clinical manifestation of CMT2J. In general, and in concordance with other mouse models for axonal CMT, the phenotype in mice is much milder than that observed in human CMT2J patients. The authors suggest that the pathomechanism of this form of CMT2 targets axonal mitochondria, causing axonal transport defects and mitochondrial disfunction leading to axonal degeneration and axonal loss over time. In future, this mouse model provides an excellent opportunity to study the exact pathological mechanisms of CMT2J and is a valuable pre-clinical model in which to test novel therapies that aim to preserve axonal integrity and function.
The experiments are very diverse and well-executed and underpin the major tenets of this study.

Minor but important point:
The gene and protein nomenclature adopted in this paper is a mess and does not conform to international standards and is furthermore inconsistent. I urge the authors to review this and make changes. For example, the gene name for Connexin32 is sometimes written as Cx32 and in

other places as Cxn32 (Cxn32 in Figure 7 but Cx32 in the text!). The MGI name for this gene is Gjb1. If the authors want to stick to Cx32, then be consistent with proteins in all capital and gene names in italics (MPZ protein, Mpz (mouse gene) or MPZ (human gene). The same goes for Kv1.1; in line 447 and 448 written like KV1.1 (first time I see it denoted like this).

As reviewer 2 suggested, we have now corrected all gene and protein nomenclatures.

The nomenclature for the T124M mutations is very colloquial. Why not adhere to accepted nomenclature? Instead of TM/+, write MPZT124M/+ (in italics and T124M in superscript: formatting lost in editorial manager). And so on….

Following the reviewer suggestion, we have now changed the nomenclature of the mutation to, $\mathit{MPZ}^{\mathrm{T124M}}$ in the text. Due to space constraints, we still use the TM/+ and TM/TM nomenclature in the figures. We hope this is acceptable.

Please review line 451: 'In general, human myelin sheaths are thin,… Rephrase.

The line (now line 624 to 625) has been rephrased to "In general, CMT2J patients myelin sheaths are thinner than healthy control, but some patients have normal or even thickened myelin sheaths".

Reviewer #3:

In this study, the authors have developed and characterized a mouse model that harbors a T124M mutation in the myelin protein zero (MPZ) with the intent of modelling CMT2J, a hereditary axonal neuropathy which is characterized by the same mutation in humans. The manuscript includes comprehensive phenotypical, electrophysiological and histological analyses of mutant mice and convincingly confirms that the T124M substitution in MPZ results in axonal neuropathy. This is important as among the many rodent models for genetically determined neuropathies, the T124M mutant closes up to the only very few ones that are known where a mutated myelin protein does not result in demyelination but primarily in progressive axonal degeneration.
As expected, no overt abnormalities in compact myelin could be observed in mutant mice. In contrast, areas of non-compact myelin appear largely disorganized, which could be explained by altered MPZ glycosylation and protein targeting. This finding is of particular interest as non-compact myelin domains are thought to constitute routes of metabolic support for axons. N-glycosylated MPZ could play a role in securing non-compact myelin integrity and hence axonal support. The authors could observe axonal degeneration and reduced levels of ATP and NAD+ in mutant mice which may hint to SARM1 mediated axonal degeneration downstream of impaired metabolic support by Schwann cells. As metabolic support by myelinating glia is one of the experimentally most challenging aspects in axo-glia interaction, no complete resolution of the malfunction at the axo-glial interface can be expected at this point and the primary axonal damage without demyelination is a finding that is striking enough in its own. However, the metabolite measurements were performed in nerve lysates and cannot unequivocally resolve the cellular source.
Overall, the manuscript is well written, the methodology employed is adequate and of high

standard, and although widely descriptive in nature, the manuscript is of high relevance to the field. However, despite these appreciations, there are certain issues that need to be addressed.

1) The source of the measured metabolites, i.e. glial vs axonal, is not resolved. NAD+, for instance, may also play a role in Schwann cells (PMID: 29921717). This limitation should be discussed.

This important point is now discussed (line 688 to 696). We agree that unfortunately the methodology used does not help in pinpointing the source of the metabolites. However, in particular for NAD+ we believe that the observed reduction in MPZ^{T124M} sciatic nerves is more likely to reflect the staus of NAD+ in axons than in SCs. Indeed, as the reviewer rightly points out, decrease of NAD+ in SC is associated with hypomyelination and SC dedifferentiation without axonal damage. However, myelin does not appear significantly altered in MPZ^{T124M} nerves. Conversely, reduction of axonal NAD+ levels have been shown to trigger axonal degeneration. The phenotypic alterations displayed by the T124M nerves in our opinion suggest an axonal specific reduction of NAD+, which coupled with mitochondrial dysfunction and slightly increased NAM levels may point to a role for SARM1 in the axonal degeneration observed in MPZ^{T124M} mice. Current studies in the lab are specifically dedicated to explore this possibility.

2) The statement "Glycolytic activity in myelinating glia is fundamental for axonal energetics and survival (45–50).", is misleading and should clearly be confined to the CNS, as it is also correctly phrased in the discussion: "However, the lactate and glycolysis pathways play a limited role in axon survival in the PNS under physiological conditions (47,67–69)."

The reviewer is correct, and we have now rephrased the sentence to specify that this is confined to the CNS.

3) The presence of ATF3-GFP in the DRG is interesting. Would the authors claim that this is the consequence of only affected myelinated sensory fibers? A comment on Remak bundle integrity (which according to the metabolite hypothesis of the authors should presumably be normal) would be helpful.

The presence of ATF3-GFP was detected in motoneurons not in DRG. As mentioned also in reply to reviewer #1, unfortunately we could not perform quantitative experiments in DRG due to very high GFP background even in the WT.

We have now added a detailed description of Remak bundles that, as the reviewer speculated, are indeed normal (line 310 to 321 and Figure S7).

4) A quantification of Schwann cell numbers and at least rough molecular characterization of the Schwann cell differentiation/de-differentiation state would add insightful information to the manuscript.

To address this point, we performed real time RT-PCR to quantify the expression of several key transcription factors that are known to regulate (positively or negatively) Schwann cell differentiation and myelination, such as SOX2, c-Jun, Id2, Oct6 and Krox20 at both 2-months (pre-

symptomatic) and 12-month (fully symptomatic). In line with the grossly normal appearance of myelin in both time points, we could not detect significant changes in the expression of these factors, confirming that the MPZ-T124M mutation does not appreciably impact on Schwann cell differentiation. These data are now discussed in the text (line 243-245) and shown in Figure S5.

5) It is apparent that the number of samples used to quantify glycolysis is far lesser than the ones used for other metabolites. Given the intrinsic variation observed, the authors cannot conclude definitively that glycolysis is not affected at all. As the authors prefer using parametric tests to assess statistical significance, the number of samples invariably affects the assessment of statistical significance.

We concur that less samples were used to measured glycolysis metabolites than for others. However, we still used at least 5 samples for each metabolites measured. Moreover, the methodology and samples used for glycolysis metabolites measurements were independent from the one used for other metabolites

Regardless, and following the reviewer's suggestion we rephrased our considerations in the text to reflect that these cannot be definitive conclusions (line 550 to 551 and - line to 664 to 666).

6) There are some major concerns in how the authors choose to perform statistical testing in experiments involving repeated measures from the same biological replicate. For instance, for the G-Ratio analysis in Fig2, the authors compare only the mean G ratios of animals even though hundreds of axons contribute to the mean, which is correct. However, in Figures 5, 6, 7 and 8, the authors compare all the measures made from all the biological replicates en masse. This results in inflated P values and the analysis has very little statistical power. To standardize this issue, I strongly suggest that the authors represent the data as Superplots (PMID: 32346721) which represent both the measures of the technical replicates (multiple measures from the same sample) as well as the resultant mean measurement of the biological replicate. The authors can then perform a Nested ANOVA that factors both the mean and the technical replicates that contribute to the mean.

Following the reviewer's recommendations, we have now used Superplots to represent results on Figures 2H, 5B, D, F, H and J, 6F, G, H, J, K, L, K, O, P, R, S, and T, 7 B and E, S7 C, D and E. Nested ANOVA were performed for each plots aforementioned.

Minor:

1. The authors need to standardize all the data plots by showing the samples. Many bar plots do not contain sample information. Also, g-ratios reflect non-continious data and shouldn't be plotted as bar charts.

As Reviewer 3 suggested, all our plots are now showing samples distribution. An extended version of g-ration analysis is now available in figure S3.

2. While performing post tests for ANOVA, the authors need to standardize the use of Tukey's or

Dunnett's. Whether they are interested in comparing the mutants to the WT animals or in comparing all the three groups needs to be standardized (See Fig1 vs all other Figures).

Following the reviewer's observation, Tukey's *post hoc* tests is now used for all analyses.

3. While representing the results of an ANOVA, the authors need to indicate the statistical significance of the ANOVA first, followed by the statistical significance of the posttest/multiple comparisons. If the ANOVA in itself does not show any statistical significance, then the posttests do not hold any statistical power by themselves. Please check the behavior ANOVA analysis provided in Fig 1.

Following this recommendation, we added ANOVA results to figure legends. Figure 1 ANOVA analysis has been corrected.

4. The raw data needs to be properly annotated so that it can be consulted without hassle. Sample and other data features like time or individual measure before averaging is missing for a lot of data sets.

Raw data are now presented and annotated in more readily understandable way.
